# 20(S)-Protopanaxdiol Suppresses the Abnormal Granule-Monocyte Differentiation of Hematopoietic Stem Cells in 4T1 Breast Cancer-Bearing Mouse

**DOI:** 10.1155/2020/8747023

**Published:** 2020-01-03

**Authors:** Wen-Qin Guo, Ying-Ge Chen, Rong-Zhen Shi, Kai He, Jian-Feng Wang, Jin-Hui Shao, Jian-Bo Wan, Jian-Li Gao

**Affiliations:** ^1^Zhejiang Chinese Medical University, Hangzhou, Zhejiang 310053, China; ^2^The First Affiliated Hospital of Medical School of Zhejiang University, Hangzhou, Zhejiang 310009, China; ^3^State Key Laboratory of Quality Research in Chinese Medicine and Institute of Chinese Medical Sciences, University of Macau, Macao, China

## Abstract

*Panax notoginseng* (PN) has been used as a *qi*- and blood-activating (*Huoxue*) drug for thousands of years in China. It has also been widely used as an anticancer drug at present. As a *Huoxue* drug, the effect of PN on hematopoietic differentiation in tumor-bearing body has been paid more and more attention. Our research found that panax notoginseng saponins (PNS), especially panaxadiol saponins (PDS) and its aglucon *20(S)-Protopanaxdiol* (PPD), could improve the immunosuppressive state by regulating the abnormal hematopoietic differentiation in a tumor-bearing body by multiple ways. An interesting phenomenon is that PDS reduced the neutrophil-lymphocyte ratio (NLR) via its inhibition effect on the granule-monocyte differentiation of spleen cells, which is associated with a decrease in the secretion of tumor MPO, G-CSF, PU.1, and C/EBP*α*. Otherwise, PDS increased the proportion of both hematopoietic stem cells and erythroid progenitor cells in the bone marrow, but inhibited spleen erythroid differentiation via inhibiting secretion of tumor EPO, GATA-1, and GATA-2. This study suggests that PNS regulated the tumor-induced abnormal granule-monocyte differentiation of hematopoietic stem cells, affecting the distribution and function of haemocytes in tumor-bearing mice.

## 1. Introduction

*Panax notoginseng* (Burk) F. H. Chen belongs to genus *Panax* of the family Araliaceae, mainly planted in Yunnan, Guangxi, and other places in China. The function of *Panax notoginseng* (PN) could be generalized as four parts to stop bleeding, blood stasis, and pain for crude drug but show blood tonic effect after produced. Therefore, *Panax notoginseng* is known as traumatology *Jinchuang* medicine and tonic food in all through the ages. At present, the application of PN is related to cardiovascular diseases [1], atherosclerosis [2, 3], and myocardial ischemia [4]. PN can reduce myocardial oxygen consumption, inhibit platelet aggregation, prolong clotting time, lower blood lipid, scavenge free radicals, and show anti-inflammatory, anti-oxidation, and also other pharmacological effects.

Accumulating reports have shown that ginsenosides, the major active component of PN, were helpful for tumor treatment [5]. As the two characteristic types of triterpenoid saponins in ginsenosides, *20(S)-protopanaxadiol saponins* (PDS) and *20(S)-protopanaxatriol saponins* (PTS), PTS holds capacity to interfere with crucial metabolism, while PDS could affect cell cycle distribution and prodeath signaling. Our research found that the aglucon of PDS can affect cell cycle distribution and apoptosis signals [6]. Lee et al.'s research demonstrated that notoginsenoside R1 (NGR1) could inhibit HCT-116 cell metastasis by suppressing migration, invasion, and adhesion through regulation of MMP-9, integrin-1, E-selectin, and ICAM-1 expression [7]. Oh et al. showed that ginsenoside Rg3 inhibits self-renewal activity of breast stem cell-like cancer cells by blocking Akt-induced HIF-1*α* activation and subsequent expression of Bmi-1 and Sox-2 [8]. Wu et al. found that ginsenoside stereoisomers 20(S)-Rg3 and 20(R)-Rg3 can suppress the growth of H22 hepatomas and enhance the level of cytokines (IL-2, IFN-*γ*) in the immune organs and serum of tumor-bearing mice without causing adverse effects [9].

The major hematopoietic organs in adults are the bone marrow, spleen, and lymph nodes. Blood cells were originated from hematopoietic stem cells (HSC) in the bone marrow, and the HSC have a pretty good differentiation function. The committed progenitor cells can be further differentiated into various progenitor cells, such as erythroid lines, monocyte-macrophage lines, megakaryocytes, and those like in [10]. Previous research studies (from other groups or our group) showed the abnormal hematopoietic stem cell differentiation in tumor occurrence and development processes [11, 12]. As an essential medicine for promoting blood circulation, nourishing the blood, and improving or eliminating asthenia syndrome in TCM (Traditional Chinese Medicine), many research studies showed that PN can improve hematopoietic cell proliferation and regulate the differentiation from HSC [13, 14]. Therefore, in this research, we supposed that PN could affect the differentiation of tumor-associated disorder of HSC, and it can influence the differentiation of myeloid and lymphoid cells.

## 2. Materials and Methods

### 2.1. Cell Culture and Animals

4T1-Luc mouse breast cancer cells were gifted by Professor Tong-Chuan He, in the University of Chicago. Cells were maintained in Dulbecco's modified Eagle's medium (DMEM) (Invitrogen, Carlsbad, CA, USA) containing 100 U penicillin/streptomycin (Genom Bio-pharmaceutical Tech, Hangzhou, China) and supplemented with 10% FBS (Sigma-Aldrich, New Jersey, USA). All cells were cultured at 37°C in 5% CO_2_ incubator.

Five-week-old female BALB/c mice were purchased from Shanghai Lab. Animal Research Center and maintained at the animal facility of Experimental Animal Research Center of Zhejiang Chinese Medical University. All procedures were performed according to protocols following the guidelines for the Use and Care of Laboratory Animals published by the Zhejiang Province (2009) and approved by the Animal Ethics Committee of Zhejiang Chinese Medical University.

### 2.2. Materials and Reagents

**Figure 1:**
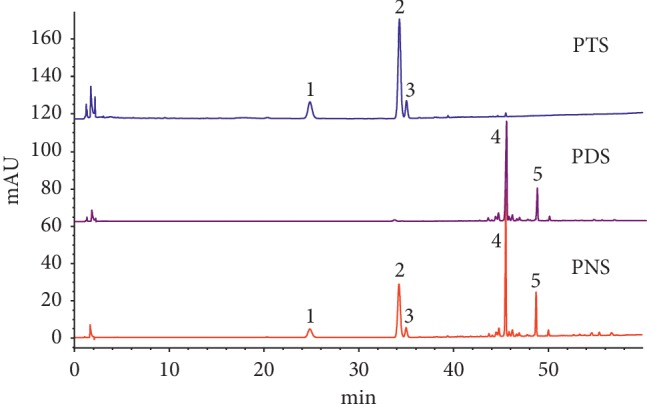
HPLC chromatograms of PTS and PDS isolated from *Panax notoginseng* saponins. Compound 1, notoginsenoside R1, 2–5, ginsenoside Rg1, Re, Rb1, and Rd, respectively.

PDS and PTS were extracted from panax notoginseng saponins (PNS), which were used in macroporous resin separation technology and used in ethanol gradient elution methods with 30%, 35%, 70%, and 80% ethanol. There are many kinds of saponins in PN, including various saponins such as notoginsenoside R1, R2, R3, R4, R5, and R6 and ginsenoside Ra, Rb, Rd, Re, and Rg1. PN with methanol and the aqueous suspension of methanolic extracts were subjected to column chromatography over amberlite XAD-2, Diaion MCI Gel HP20, or Kogel BG4600. After removing saccharides and amino acids with water, the columns were eluted with methanol to obtain a saponin fraction. Those ginseng saponins and ginsenoside Rx were separated on HPLC. PTS mainly contains triol type saponins (R1, Rg1, and Re), and PDS mainly contains glycol-type saponins (Rb1 and Rd) (Figure 1). Among them, notoginsenoside R1, ginsenoside Rg1, and Rb2 have a strong anticancer effect both *in vitro* and *in vivo* [15]. PPD was purchased from Shanghai Tongtian Biotechnology Co., Ltd. Other reagents were obtained from Sigma-Aldrich.

### 2.3. 4T1 Tumor-Bearing Mice Model

The mice were injected 10^6^ 4T1-Luc cells on the right side of the second pair of breast fat pads to prepare mouse 4T1 breast cancerous tumor model [16]. Before injection, cells were resuspended in PBS and analyzed by 0.4% trypan blue exclusion assay (viable cells, >90%). At 48 h after tumor cell injection, PNS, PDS, and PTS were administered 20 mg/kg body weight to mice every day, G-CSF was administered 30 *μ*g/kg body weight to mice every day, EPO was administered 60 U/kg body weight to mice every day, and CTX was administered 30 mg/kg body weight to mice once in every 2 days for 25 days.

The diameter *L* (mm) and the short diameter *W* (mm) of the tumor were measured every 5 days after tumor implant. Tumor volume was calculated using the following equation: V = (*L* + *W*) × (*L*) × (*W*) × 0.2618 [17].

After the mice were sacrificed, the thymus, spleen, and tumor were dissected and weighed to calculate the organ index: organ index (g/g) = organ weight (g)/body weight (g) × 100%. The inhibition rate of tumor growth was evaluated by the following formula: inhibition rate of tumor growth (%) = (1 − (average tumor weight of administrartion group/average tumor weight of the model group)) × 100%.

### 2.4. Animal Bioluminescence Imaging and Blood Routine Analysis

Animal bioluminescence imaging was carried out as described previously [18, 19]. At the 3rd week of administration, mice were anesthetized with isoflurane attached to a nose-cone mask equipped with a Xenogen IVIS 200 imaging system (Caliper Life Sciences, Hopkinton, MA). For imaging, mice were injected intraperitoneally with 5 mg/mL D-luciferin sodium salt (Gold Biotechnology, St. Louis, MO) at 100 mg/kg body weight in 0.1 ml of sterile PBS. Acquired images were obtained by superimposing the emitted light over the grayscale photographs of the animal. Quantitative analysis was done with Xenogen's Living Image V2.50.1 software as described previously [20].

Mouse peripheral blood (100 *μ*L) was collected into tubes containing EDTA (Genom Bio-pharmaceutical Tech, Hangzhou, China) before sacrifice. Routine blood tests were immediately performed using a Sysmex XT-2000i automated hematology analyzer (Sysmex Corp., Hyogo, Japan) for the number of red blood cells (RBC), white blood cell (WBC), hemoglobin (HGB), and hematocrit (HCT). Blood smears from a handful of specimens from mouse were prepared in the slide glass and processed for leukocyte differential staining (Wright's stain).

### 2.5. XTT Assay for the Toxicity of PPD on Mouse Spleen Cells

A modified XTT assay was used to examine the cell proliferation as described [21]. Briefly, mouse spleen cells (5 × 10^4^/well) were seeded in 96-well plates. PPD were added to the cells at variable concentrations or solvent control (0.1% DMSO). At 24 h after treatment, 50 *μ*L XTT dyesolution was added to each well and incubated for additional 4 h. Absorbance at 450 nm was measured using a 96-well microplate reader (Bio-TeR, Power wave 340, USA).

### 2.6. Histological Evaluation and Immunohistochemical (IHC) Staining

Retrieved thymus and spleen tissues were fixed in 10% formalin and embedded in paraffin. Serial sections of the embedded specimens were stained with hematoxylin and eosin (H&E) for thymus tissue. On the other hand, immunohistochemical staining was carried out for spleen tissue. Slides were deparaffinized and then rehydrated in a graduated fashion [22], and the deparaffinized slides and fixed 4T1 cells were subjected to antigen retrieval. There into, 4T1 cells were probed with anti-EPO and G-CSF antibodies, spleen tissue were marked with MPO antibody (Santa Cruz, CA, USA), and isotype IgG control was used as negative control. The slides and cells were followed by incubation with biotin secondary antibodies and streptavidin-horseradish peroxidase. The presence of the expected protein was visualized by DAB staining and examined under a microscope (Motic China Group Company, Shanghai, China; magnification, ×400).

### 2.7. Colony-Forming Assay of BFU-E, CFU-E, and CFU-GM

Splenocyte cells were isolated from normal or tumor-bearing mice, resuspended in RPMI 1640 with 2% FBS, and counted. Then, 5 × 10^4^ nucleated splenocytes were plated in methyl cellulose media, and PPD were divided into three dose groups (0.1, 1, 10 *μ*M), cultured in a methyl cellulose medium, and placed in a 37°C, 5% CO_2_ cell incubator. After 10 days of culture, BFU-E, CFU-E, and CFU-GM colonies were counted under a microscope (Olympus, IX71, Japan).

### 2.8. Flow Cytometry Analysis

Isolated spleen cells were cultured in methylcellulose for 12 days; all cells were resuspended in 200 *μ*L PBS, fixed with 70% ethanol for 24 h, washed with PBS, and blocked with 1% BSA for 20 minutes in 4°C. And then, they were incubated with FITC-CD71 and PE-Ter119 (erythrocyte), FITC-CD34 (HSCs), or FITC-CD11b and PE-Gr-1 antibodies (BD Pharmingen, New Jersey, USA) (monocytes or granulocyte) for 30 min on the ice in dark. Data were acquired on a Guava easy Cyte 6-2L and analyzed with a FCS express V3 (Guava Technologies, Hayward, CA, US).

### 2.9. RNA Isolation and Quantitative PCR Analysis

Spleen cells were isolated from normal mouse and 4T1 tumor-bearing mouse. Cells were maintained in Roswell Park Memorial Institute (RPMI) containing 100 U penicillin/streptomycin and supplemented with 20% FBS and treated with PPD at various concentrations (0.1, 1, and 10 *μ*M) for 48 h. The total RNA of all spleen cells was extracted with Trizol (Invitrogen), and purity and concentration of the extracted RNA were measured on a UV spectrophotometer. Then, cDNA was synthesized by reverse transcription, and fluorescence quanititavie dectection of the target gene was performed afterwards. All reactions were performed in a thermal cycler (ABI7500, ABI, USA) with primers (Sangon Biotech, Shanghai, China) for GADPH (glyceraldehyde-3-phosphate dehydrogenase), forward: 5′- GGC TGC CCA GAA CAT CAT -3′ and reverse: 5′- CGG ACA CAT TGG GGG TAG -3′; PU.1(Spi-1 proto-oncogene), forward: 5′- CCT GTA TGT AGC GCA AGA TTT A-3′ and reverse: 5′- AAT GTT CCA GTT GGG TCC AG-3′; GATA-1 (GATA binding protein 1), forward: 5′- CCA GCA CAA GTT CCT GAT TTT ATC -3′ and reverse: 5′- GTC CTT CGG CTG CTC CTG TG -3′; C/EBP*α* (CCAAT/enhancer binding protein, alpha), forward: 5′- ACG TGG AGA CGC AGC AGA A-3′ and reverse: 5′- GTA GGC ATT GGA GCG GTG A-3; GATA-2 (GATA binding protein 2), forward: 5′- TAA CAG GCC ACT GAC CAT GA-3′ and reverse: 5′- GAT AGG CGT TGG CGT AGG TA-3′; and GM-CSF (Granulocyte-macrophage colony-stimulating factor), forward: 5′- GGC CTT GGA AGC ATG TAG AGG-3′ and reverse: 5′- GAA CTC GTT AGA GAC GAC TT -3′. The results of RT-PCR were analyzed, that is, the Ct values of each sample were analyzed, the 2^−ΔΔCT^ values of each target gene were calculated, and the differences in the expression levels of different groups of spleen cells were obtained.

### 2.10. Statistical Analysis

All values are expressed as the means ± standard deviation of four measurements. Statistical analysis was performed using a *t*-test and two-tailed distribution, assuming two-sample unequal variance. A *P* < 0.05 or *P* < 0.01 value was considered statistically significant.

## 3. Results

### 3.1. PDS, PTS, and PNS Protected the Structure of Spleen and Thymus and Inhibited Breast Cancer Growth and Metastasis

**Figure 2:**
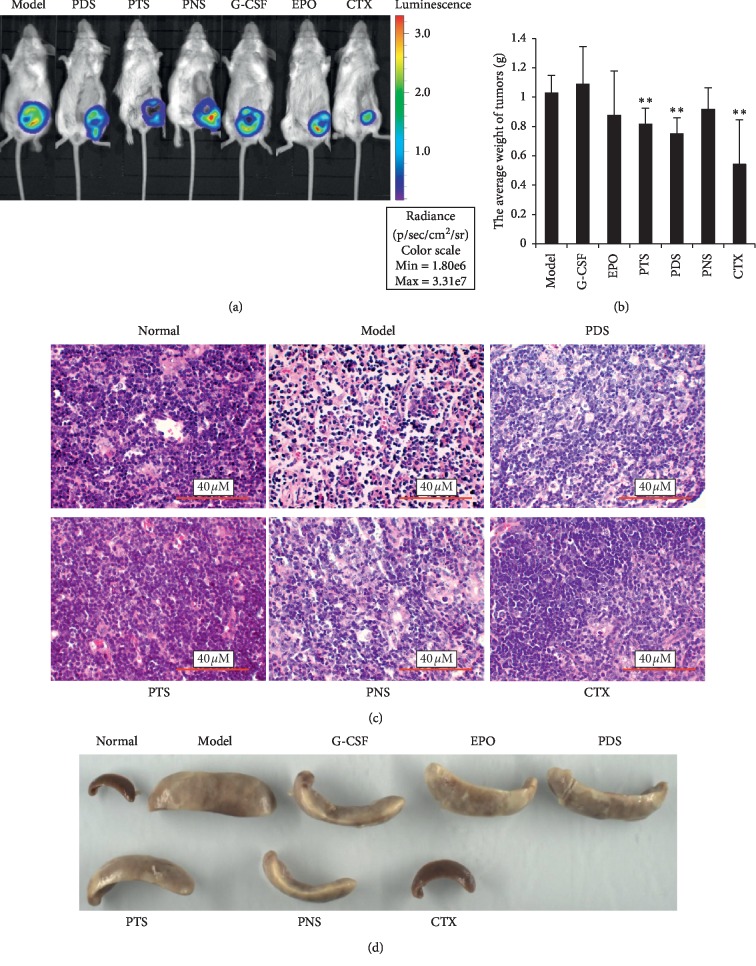
(a) Xenogen images of the representative mouse in the model group, PDS, PTS, and PNS group. (b) The average tumor weight of mice, ^*∗∗*^*P* < 0.01, in PDS, PTS, and PNS groups vs. the model group. (c) Hemotoxylin & Eosin staining of mice thymus tissue (×400 magnification). (d) The changes of spleen after PDS, PNS, and PDS administration.

PDS exhibited antitumor proliferation effect on 4T1 tumor-bearing mice after 3 weeks of treatment *in vivo* (Figures 2(a) and 2(b)). As shown in Figure 2(a), animals in the model group have higher signal compared with the PTS and PNS group, which suggested that PTS and PNS inhibited 4T1 breast tumor growth in a mouse. Meanwhile, the average weight of tumors was decreased by PDS treatment, and the average tumor weight is 1.029 ± 0.118 g in the model group and 0.749 ± 0.110 g in PDS group (*P* < 0.05). On the other hand, the earliest occurrence of lung metastasis was in EPO and model groups, and the mice in PTS, PNS, and CTX groups developed metastases to lung later than the model group. Otherwise, PDS, PTS, and PNS at the doses used were not toxic to the animals as we observed no differences in animal body weights and behaviors.

As shown in Table 1, compared with the normal group, the spleen index of the model group was higher, and there was a significant difference (*P* < 0.05). Compared with the model group, the tumor index and spleen index of the CTX group were lower, the spleen index of G-CSF group was lower, and there was a significant difference (*P* < 0.05). The tumor index of the PDS group was lower, the lung index was higher, and there was a significant difference (*P* < 0.05). We can observe the changes in spleen after PDS, PNS, and PDS administration, especially PNS groups of the spleen were smaller than those of the model group (Figure 2(d)).

The results of HE staining showed that the structure of thymus cells in the model group was disordered, while the thymocyte structure in the PTS and PDS group was significantly better than that in the model group, and the obvious dark areas in the open area was significantly higher than that in the model group. It was also observed that the density of small lymphocyte clusters and lymphocytosis in the PDS group is higher than that of the model group (Figure 2(c)).

### 3.2. PDS, PTS, and PNS Regulate the Hematopoietic Capacity and Hematopoietic Differentiation in Tumor-Bearing Mice

**Figure 3:**
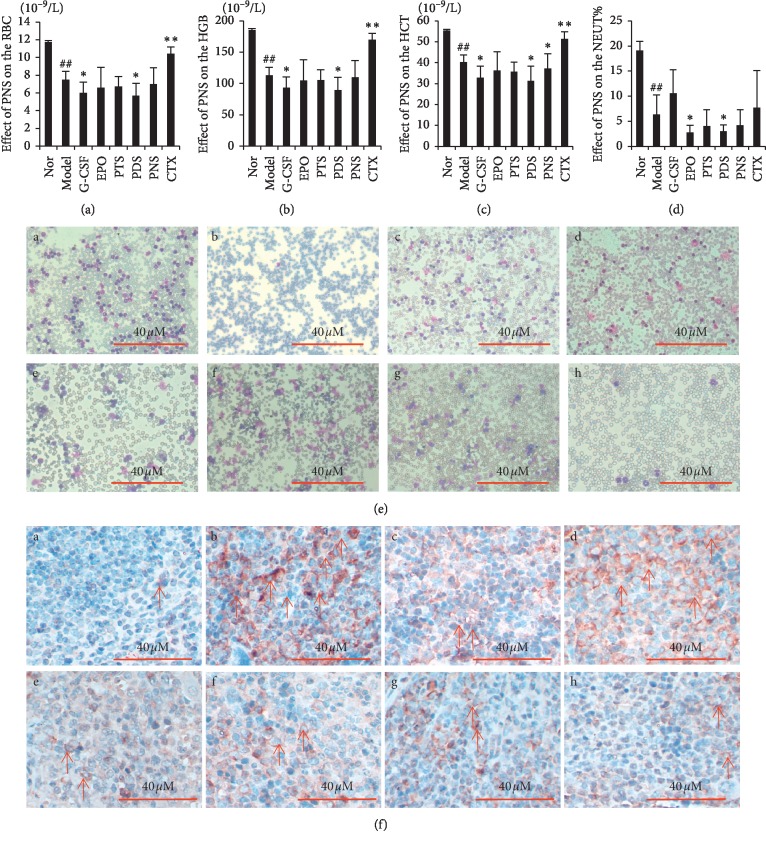
PDS, PTS, and PNS promote the hematopoietic capacity of the tumor-bearing mice. (a–d) The RBC, HGB, HCT, and NEUT% in mouse peripheral blood, ^*∗*^*P* < 0.05,^*∗∗*^*P* < 0.01 vs. model group. *##P* < 0.01, vs. normal group. (e) Morphological pictures of peripheral blood for different groups. (f) Immunohistochemical staining of mice spleen tissues. MPO expression in mice spleens (×400 magnification). a: normal group; b: model group; c: EPO group; d: G-CSF group; e: PDS group; f: PTS group; g: PNS group; and h: CTX group.

Some previous research reported the tumor-associated acute anemia in mice [12, 23, 24]; in this study, we found that PDS-treated mice showed alleviated anemia than the model group (Figures 3(a)–3(d)), and the RBC, HGB, and HCT in mouse peripheral blood were decreased (RBC: 7.50 ± 0.95 ∗ 10^−9^/L in the model group vs. 5.70 ± 1.41 ∗ 10^−9^/L in the PDS group, *P* < 0.05; HGB: 113.17 ± 12.58 ∗ 10^−9^/L in the model group vs. 89.17 ± 20.70 ∗ 10^−9^/L in the PDS group, *P* < 0.05; HCT: 40.18 ± 3.51 ∗ 10^−9^/L in the model group vs. 31.18 ± 7.24 ∗ 10^−9^/L in the PDS group, *P* < 0.05).

The NEUT% in mouse peripheral blood was decreased, 6.33 ± 3.93% in the model group vs. 2.97 ± 1.30% in the PDS group, *P* < 0.05. Morphological pictures of peripheral blood also showed the increase of monocytes and lymphocytes, and reduction of erythrocyte in the model group, PNS, and PDS both show obvious therapeutic effects, and PTS, PNS, and PDS significantly reduced neutrophils in breast cancer-bearing mice (Figure 3(e)).

Otherwise, the number of hematopoietic stem cells and erythroid progenitor cells were increased in the bone marrow, and monocytes were decreased in the PTS and PDS group (CD34^+^: 2.22 ± 0.32% in the model group vs. 4.42 ± 1.11% in the PTS group, *P* < 0.05; CD71^+^/Ter119^+^: 0.17 ± 0.10% in the model group vs. 0.33 ± 0.30% in PDS, *P* > 0.05; and CD11b^+^/Gr-1^+^: 3.75 ± 3.19% in the model group vs. 72.04 ± 7.13%, 77.53 ± 5.18%, 75.20 ± 5.86% in the PDS, PTS, and PNS group, *P* < 0.05). It indicated that the bone marrow erythroid was inhibited and the differentiation of monocytes was decreased in breast cancer-bearing mice.

### 3.3. PDS and PTS Inhibits MPO Expression in the Spleen of Tumor-Bearing Mice

MPO expression was confirmed in spleen tissues by immunohistochemistry of paraffin embedded spleen slides. Granulocytes and their precursors are expressed as MPO positive, and we found out that the expression of MPO in PDS, PTS, and PNS groups was lower than that in model group (Figure 3(f)). Neutrophils, monocytes, and some macrophages secreted MPO, so the result indicated that PDS and PTS can decrease spleen hematopoietic stem cells granule-monocyte differentiation.

### 3.4. Effect of PPD on the Colony Formation of Spleen Cell of Tumor-Bearing Mice

**Figure 4:**
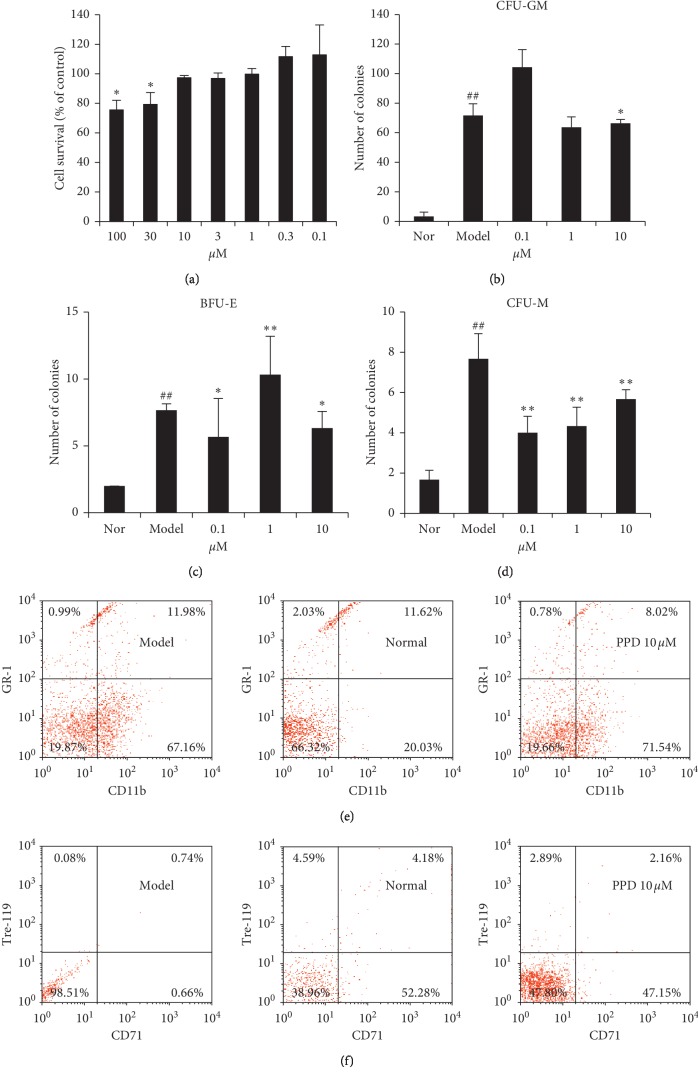
(a) The cytotoxicity effect of PPD in mouse splenocytes.^*∗*^*P* < 0.05, vs. normal group. (b–d) The effect of PPD on the colon formation of BFU-E, CFU-M, and CFU-GM, ^*∗*^*P* < 0.05, ^*∗∗*^*P* < 0.01, vs. model group. *##P* < 0.01, vs. normal group. (e-f) The proportion of monocyte (CD11b^+^/Gr-1^–^), granulocyte (CD11b^+^/Gr-1^+^), and erythroid progenitor cells (CD71^+^/Ter119^+^) in mouse splenocytes.

PDS could be absorbed in the gastrointestinal tract following oral intake of ginsenosides which are partly transformed into the PPD through a series of deglycosylation procedures by acid hydrolysis and intestinal bacterial actions [25]. XTT results showed that PPD 100 *μ*M and 30 *μ*M significantly inhibited the proliferation of mouse spleen cells, while 10 *μ*M or lower did not inhibit proliferation of mouse spleen cells (Figure 4(a)).

Spleen hematopoietic stem cells can differentiate into erythrocyte bursting colony-forming units (BFU-E), granulocyte-macrophage colony-forming units (CFU-GM), and macrophage colony-forming cells (CFU-M). *In vitro*, 0.1, 1, and 10 *μ*M PPD were used to analyse the colony formation ability of the PPD in the mouse spleen cells. As a result, PPD 0.1 *μ*M, 1 *μ*M, and 10 *μ*M could inhibit BFU-E and CFU-M than controls (Figures 4(b), 4(c), *P* < 0.05), and PPD 1 *μ*M and 10 *μ*M could inhibit CFU-GM compared with controls (Figure 4(d), *P* < 0.05).

### 3.5. PPD Changed the Proportion of Erythroid and Myeloid Progenitor Cells in Spleen Cells

The expression of CD11b^+^/Gr-1^−^ and CD71^+^/Ter119^+^ in mouse spleen cells were significantly decreased compared with that of controls (CD11b^+^/Gr-1^−^: 58.94 ± 8.83% in control vs. 44.44 ± 4.88% in the PPD 10 *μ*M group, *P* < 0.05; CD71^+^/Ter119^+^: 1.40 ± 0.69% in control vs. 0.59 ± 0.05% in the PPD 10 *μ*M group, *P* < 0.05) (Figures 4(e) and 4(f)).

### 3.6. Effect of PPD on Erythroid and Myeloid Differentiation-Related Gene Expression in Spleen Cells of Tumor-Bearing Mice

**Figure 5:**
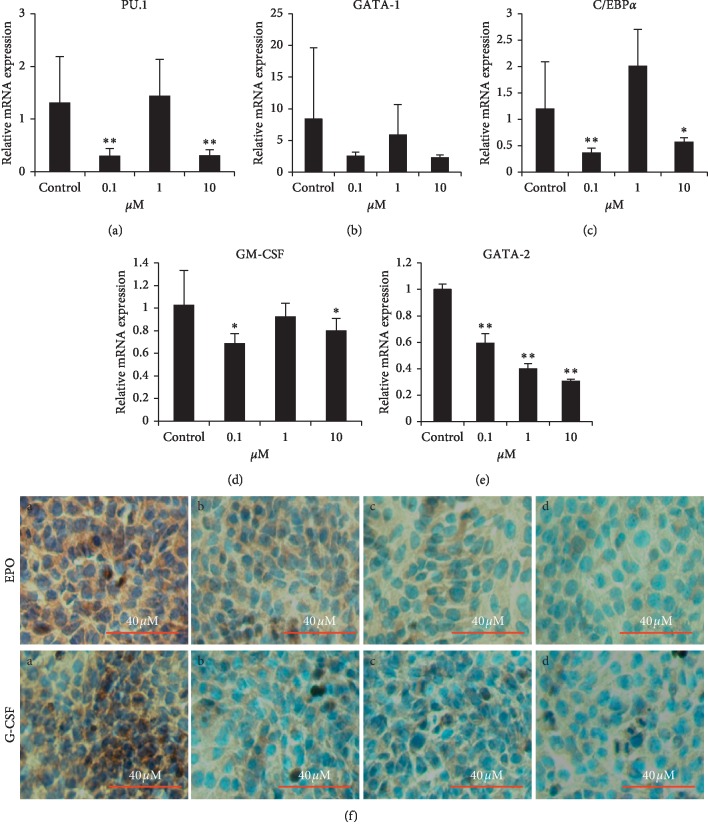
(a–e) The relative expression of C/EBP*α*, GATA-1, GM-CSF, GATA-1, and PU.1. Data are from three independent experiments. Values are shown as means ± SD (*n* = 3). ^*∗*^*P* < 0.05, ^*∗∗*^*P* < 0.01 vs. control. (f) EPO and G-CSF expression in 4T1 cells. PPD reduced expression of G-CSF and EPO in 4T1 (×400 magnification). a: control; b: PPD 0.1 *μ*M; c: PPD 10 *μ*M; d: PPD 100 *μ*M.

GATA-1 and GATA-2 play key roles in erythroid differentiation of hematopoietic stem cells, and GATA-1 and GATA-2 were reduced than those in controls. PU.1, C/EBP*α*, and GM-CSF were important in the differentiation of the myeloid, monocytic, myeloid, and lymphatic system, in which mRNA expression levels were reduced than those of controls (Figures 5(a)–5(e)).

### 3.7. Effect of PPD on EPO and G-CSF Secretion in 4T1-Luc Cells of Tumor-Bearing Mice

PPD was administrated for 48 h, and the expression of EPO and G-CSF in 4T1-Luc cells was detected by immunocytochemical staining. The results showed that the expressions of EPO and G-CSF in the PPD-treated group were lower than that in controls (Figure 5(f)).

## 4. Discussion

Many studies have shown the application of PNS in the treatment of gastric cancer, liver cancer, breast cancer, and other malignancies [7, 26–30]. PNS can directly inhibit tumor cell growth or metastasis, induce tumor cell apoptosis and reverse the multidrug resistance of tumor cells, and stimulate the immune function of the body. PNS, PDS, and PTS, as well as the main monomers (ginsenosides Rg1 and Rb1) can be inhibited in TNF-*α*-induced vascular inflammation, with PDS exhibiting the strongest anti-inflammatory activity [31]. Also, recent research revealed the antitumor mechanism of ginsenoside is that PPD was confirmed to have a low KD of 1.22 *μ*M when interacting with the Ras protein, and the mutation sites of G12 A and G60 demonstrated core roles in those interactions. [32].

In our study, PDS showed an antibreast cancer effect by delaying invasion and metastasis in tumor-bearing mice. It could decrease the number of RBC, HGB, and HCT in tumor-bearing mice. We found that PDS increased the proportion of both hematopoietic stem cells and erythroid progenitor cells in the bone marrow, but PPD inhibited spleen erythroid differentiation and reduced the expression of GATA-1 and GATA-2 and could not improve tumor-induced anemia in mice. The inhibition of erythroid differentiation by PPD may be associated with the decreased secretion of tumor erythropoietin (EPO) and the subsequent reduced expression of GATA binding protein 1 (GATA-1) and GATA binding protein 2 (GATA-2). GATA-1 and GATA-2 are widely involved in the development of hematopoietic cells in the GATA family. Yu et al. showed that GATA-2 is already expressed in early embryos, while GATA-1 is upregulated with the further differentiation and maturation of erythroid progenitor cells [33]. Furthermore, Castaño et al. found that GATA-2, at the mesoderm specification stage, promotes the generation of hemogenic endothelial progenitors and their further differentiation to hematopoietic progenitor cells and negatively regulates cardiac differentiation [34]. EPO is also involved in erythropoiesis and is an important factor that supports the terminal differentiation of erythroid cells, in animal models, and modulates the sensitivity of the erythroid cells to EPO.

PDS reduced the spleen index but increased the thymus index in tumor-bearing mice and reduced the NEUT%, NLR, and MPO expression of tumor-bearing mice. This activity of PDS may be related to its inhibition effect on spleen cell granule-monocyte differentiation, which is associated with a decrease in the secretion of tumor G-CSF and the subsequent reduce expression of PU.1 and C/EBP*α*. The NLR is an indicator of systemic inflammation and has been associated with the survival of many types of cancer [35]. The increase in granulocyte differentiation will directly lead to the increase in NLR. G-CSF stimulates the HSCs differentiation into mature neutrophilic granulocytes (neutrophils) in the bone marrow (BM) [36]. It is a hemopoietic growth factor that has a role in steady state granulopoiesis, as well as in mature neutrophil activation and function.

C/EBP*α* is an essential transcription factor for myeloid lineage commitment. House et al. found that C/EBP*α* has the effect of inhibiting cell proliferation and promoting cell differentiation, and it is mainly expressed in granulocytes, monocytes, and hepatocytes [37]. PU.1 is a specific hematopoietic member of the Ets family and is a key factor in the regulation of myeloid gene expression. Experiments have shown that the transactivation function of PU.1 can be inhibited by GATA‐1 and GATA‐2 through direct protein interactions [38]. PPD could reduce the expression of PU.1 and C/EBP*α*, indicating that PPD can inhibit spleen cell granule-monocyte differentiation by reducing expression of PU.1 and C/EBP*α*.

In conclusion, in this research, we evaluated the impact of PDS, and its acid hydrolysis produces PPD to regulate the hematopoietic stem cell differentiation in a 4T1 breast cancer-bearing mouse. We found that the anticancer effect of PDS may be related to its inhibition effect on spleen cell granule-monocyte differentiation, which is associated with a decrease in the secretion of G-CSF and MPO and the subsequent reduction in the expression of PU.1 and C/EBP*α*. Further research in this topic will open up new fields for understanding tumor-induced abnormal hematopoietic stem cells differentiation and for identifying potential therapy strategies for cancer.

## Figures and Tables

**Table 1:** Effect of PDS, PTS, and PNS on tumor inhibition rate and organic coefficient (g/100 g) in breast cancer mouse (*n* = 6).

	Tumor (g/100 g)	Spleen (g/100 g)	Lung (g/100 g)	Tumor inhibition rate	Metastatic rate
Normal group		1.00 ± 0.05	1.855 ± 0.109		—
Model group	5.07 ± 0.78	7.69 ± 0.67^△△^	1.459 ± 0.726		3/6
G‐CSF group	5.61 ± 1.66	6.86 ± 0.51^*∗*^	1.989 ± 0.713	−5.80%	3/6
EPO group	4.42 ± 1.08	7.33 ± 0.88	2.127 ± 0.401	14.80%	2/6
PTS group	4.40 ± 1.20	7.56 ± 1.75	1.770 ± 1.039	20.80%	1/6
PDS group	4.05 ± 0.97^*∗*^	7.10 ± 0.47	2.317 ± 0.383^*∗*^	27.20%	3/6
PNS group	4.52 ± 0.74	6.91 ± 0.97	2.439 ± 0.140^*∗*^	10.80%	1/6
CTX group	3.06 ± 1.73^*∗*^	2.49 ± 0.75^*∗∗*^	1.889 ± 0.244	47.30%	0/6

^Δ^ *P* < 0.05, ^ΔΔ^*P* < 0.01 vs. normal group; ^*∗*^*P* < 0.05, ^*∗∗*^*P* < 0.01 vs. model group.

## Data Availability

The data used to support the findings of this study are available from the corresponding author upon request.

## Abbreviations

BFU-E: Burst-forming unit-erythroid
C/EBP*α*: CCAAT/enhancer binding protein alpha
CFU-GM: Colony-forming unit-granulocyte, macrophage
CFU-M: Colony-forming unit-macrophage
DMEM: Dulbecco's modified eagle's medium
DMSO: Dimethyi sulfoxide
EPO: Erythropoietin
FBS: Fetal bovine serum
GATA-1: GATA binding protein 1
GATA-2: GATA binding protein 2
G-CSF: Granulocyte colony-stimulating factor
GM-CSF: Granulocyte-macrophage colony-stimulating factor
HCT: Hematocrit
HGB: Hemoglobin
HSC: Hematopoietic stem cells
NEUT%: Neutrophil percentage
NGR1: Notoginsenoside R1
NLR: Neutrophil-lymphocyte ratio
PDS: 20(S)-Protopanaxadiol saponins
PLT: Platelet
PNS: Panax notoginseng saponins
PPD: 20(S)-Protopanaxdiol
PTS: 20(S)-Protopanaxatriol saponins
PU.1: Spi-1 proto-oncogene
RBC: Red blood cells
RPMI: Roswell Park Memorial Institute
TCM: Traditional Chinese Medicine
WBC: White blood cell
XTT: XTT sodium
CTX: Cyclophosphamide.
